# Temporal Associations of the SARS-CoV-2 NP Antigen and Anti-Spike Total Ig Levels with Laboratory Parameters in a Greek Cohort of Hospitalized COVID-19 Patients

**DOI:** 10.1155/2021/6590528

**Published:** 2021-09-20

**Authors:** Erasmia Rouka, Ourania S. Kotsiou, Garyfallia Perlepe, Athanasios Pagonis, Ioannis Pantazopoulos, Konstantinos I. Gourgoulianis

**Affiliations:** ^1^Department of Respiratory Medicine, Faculty of Medicine, University of Thessaly, BIOPOLIS,41110, Larissa, Greece; ^2^Nursing Department, School of Health Sciences, University of Thessaly, GAIOPOLIS,41110, Larissa, Greece

## Abstract

**Background:**

The direct effect of SARS-CoV-2 on the lungs results in increased hospitalization rates of patients with pneumonia. Severe COVID-19 patients often develop ARDS which is associated with poor prognosis. Assessing risk factors for COVID-19 severity is indispensable for implementing and evaluating therapeutic interventions. We investigated the temporal associations between the SARS-CoV-2 antigen (Ag), total Immunoglobulin (Ig) levels, and several laboratory parameters in hospitalized patients with varying degrees of COVID-19 severity.

**Methods:**

The SARS-CoV-2 nucleocapsid protein (NP) and total Ig Spike (S) protein-specific antibodies were determined for each patient with lateral flow assays through repeated sampling every two days. Hematological and biochemical parameters were evaluated at the same time points.

**Results:**

40 Greek COVID-19 patients (31 males, 9 females) with a median age of 59.50 ± 16.21 years were enrolled in the study. The median time from symptom onset to hospitalization was 8.0 ± 4.19 days. A significant negative correlation was observed between the SARS-CoV-2 Ag and total Ig levels. The temporal correlation patterns of the SARS-CoV-2 NP Ag and anti-S total Ig levels with laboratory markers varied among patients with differing degrees of COVID-19 severity. Severe-critical cases had lower SARS-CoV-2 Ag and increased total Ig levels as compared to mild-moderate cases.

**Conclusions:**

Distinct temporal profiles of the SARS-CoV-2 NP Ag and anti-S total Ig levels may distinguish different groups of COVID-19 severity.

## 1. Introduction

The coronavirus disease 2019 (COVID-19), caused by the severe acute respiratory coronavirus 2 (SARS-CoV-2), leads to high rates of hospitalizations due to pneumonia [1]. A major determinant of high mortality in severe COVID-19 patients is the acute respiratory distress syndrome (ARDS) secondary to viral pneumonitis [2]. A systematic review and meta-analysis of 28 studies reported that the overall pooled mortality estimate among 10.815 ARDS cases in COVID-19 patients was 39% [2]. Alveolar damage and pulmonary microvascular thrombosis are thought to be the major causes of acute lung injury in COVID-19, yet the underlying mechanisms of SARS-CoV-2-related coagulopathy are still under study [3].

Risk factors for COVID-19 severity and mortality have been in the focus of research as requisites for the identification of cohorts at high risk and the evaluation of therapeutic interventions. SARS-CoV-2 viral load (especially plasma viraemia) has been associated with increased respiratory disease severity, systemic inflammation, and mortality [4]. However, it has also been reported that severely ill patients produce very high anti-SARS-CoV-2 antibody titres and have lower viral load as compared to those with mild disease [5]. Inconsistent results regarding the viral load and antibody dynamics during the course of SARS-CoV-2 could be attributed to the considerable heterogeneity of assays used, population characteristics, and outcomes measured [6].

Greece has suffered 12.935 deaths among 487.709 confirmed cases over a period of 18 months (https://covid19.who.int/region/euro/country/gr). Studies focusing on factors affecting the severity of COVID-19 in Greek patients are limited [7, 8]. In addition, consecutive data to investigate these factors have not been collected. Serial measurements of virus- and host-associated variables are important for understanding the disease course of SARS-CoV-2 infection. In addition, knowledge of the viral and host dynamics during hospitalization is key to optimizing therapeutic interventions. We investigated the temporal associations of the SARS-CoV-2 nucleocapsid protein (NP) and anti-Spike (S) total Immunoglobulin (Ig) levels with clinical and laboratory parameters in hospitalized patients with varying degrees of COVID-19 severity.

## 2. Materials and Methods

### 2.1. Subject Enrollment and Study Design

We prospectively studied 40 patients with real-time reverse-transcription polymerase chain reaction- (RT-PCR-) confirmed SARS-CoV-2 infection who were admitted to the COVID-19 Department of the University Hospital of Larissa, Greece, from December 6th, 2020, to January 17th, 2021. The subjects were followed up until discharge from the COVID-19 department or death. Follow-up was concluded on January 30, 2021. The study was approved by the Research Ethics Committee of the University Hospital of Larissa (36660/21.09.2020), and written informed consent was obtained from each subject involved. Patients' demographic and baseline characteristics including smoking and past and current medical history were documented. Time from symptom onset to hospitalization was also recorded. COVID-19 severity was defined according to the Chinese management guidelines (released on March 3, 2020) [9]. Patients were divided into two groups (mild-moderate cases; severe-critical cases).

### 2.2. Laboratory Investigation

Nasopharyngeal swab and whole blood specimens were collected from each patient every two days since admission, for the semiquantitative determination of the SARS-CoV-2 NP antigen (Ag) and the semiquantification of the IgA, IgM, and IgG (total Ig) spike protein-specific antibodies, respectively, with lateral flow immunochromatographic assays (Catalog numbers: V1310/1330 and V1210/V1230, Prognosis Biotech, Larissa, Greece). This technology has been previously used by our group and the method description partly reproduces our wording [10].

A control line served as a procedural control for the assays used. The absence of a colored line in the control region was indicative of an invalid test result. In the case of the SARS-CoV-2 Ag lateral flow immunoassay, an initial dilution of 1000 ng NP per ml running buffer was made, followed by 6 serial dilutions. These dilutions were used as standards. In each standard, 10 lateral flow strips were used. At the end of the test's procedure (after 15 minutes), the test strips were scanned in the S-flow reader. The scanner has the ability to measure the density of the test line and the control line and to automatically create their ratio (T/C). In a similar manner, 8 standards of recombinant antibodies were used in order to create the standard/ratio curve for the anti-spike total Ig semiquantification.

According to the manufacturer, clinical performance with 528 nasal specimens (prior confirmed with RT-PCR assay) showed 98.59% sensitivity and 99.74% specificity for the Rapid Test Ag 2019n-CoV (https://www.prognosis-biotech.com/products/covid-19/rapid-test-ag-2019-ncov/) while the clinical diagnostic sensitivity and specificity for the Rapid Test 2019-nCoV Total Ig were 98.75% and 100%, respectively (https://www.prognosis-biotech.com/products/covid-19/rapid-test-2019-ncov-total-immunoglobulins/).

Additional blood and serum samples were collected on the same dates for the evaluation of hematological and biochemical parameters: blood count, D-dimer (D-D), ferritin, C-reactive protein (CRP), lactate dehydrogenase (LDH), lactic acid, urea, creatinine, and alanine transaminase (ALT).

### 2.3. Statistical Analysis

Statistical analysis was performed using the SPSS v 19.0 software (IBM). The Kolmogorov–Smirnov normality test was used to assess data distribution. Associations between quantitative variables were evaluated with the Pearson (*r*) or the Spearman (*ρ*) correlation coefficients as appropriate. The Mann–Whitney test was used to assess significant differences of continuous nonparametric data between the two groups of patients. The chi-square test was used to determine associations between categorical variables. Statistical significance was set at the *p* < 0.05 level.

## 3. Results

### 3.1. Baseline Characteristics of the Study Population

The demographic and clinical characteristics of the study population are presented in Table 1. The most common complication at admission was pneumonia followed by acute kidney damage. Out of 40 patients, 30% (12) were grouped as mild-moderate cases and 70% (28) were classified as severe-critical cases. Within the latter group, 3 subjects were designated as critically ill. All subjects had at least one coexisting comorbidity. Length of stay in the COVID-19 Department ranged from 4 to 16 days. Total inpatient days were in the range of 4 to 60. The 3 patients with critical COVID-19 died by the end of the study period. The remaining subjects were discharged home or to rehabilitation centres.

### 3.2. Correlation between the SARS-CoV-2 NP Ag and Anti-S Total Ig Antibody Levels with Laboratory Biomarkers

**Figure 1:**
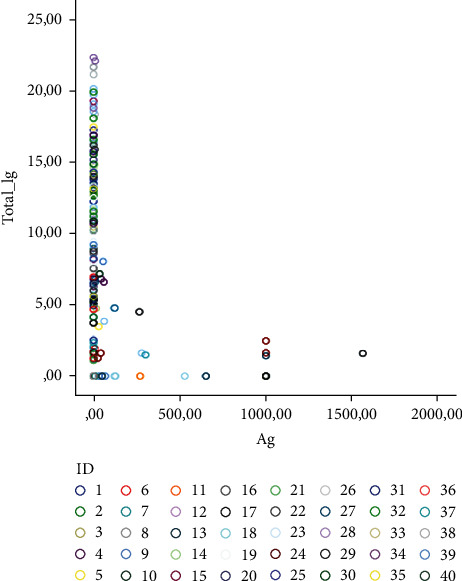
Scatter plot of all Ag-total Ig values. Markers have been set by case ID.

The mean values of all laboratory data are shown in Table 2. The scatter plot of all Ag-total Ig values is presented in Figure 1. In the initial pooled analysis, a significant negative correlation was observed between the SARS-CoV-2 Ag and total Ig levels (*ρ* = −0.489/*p* < 0.001). SARS-CoV-2 Ag levels were inversely correlated with ALT (*ρ* = −0.408/*p* < 0.001), white blood cells (WBC) (*ρ* = −0.227/*p* = 0.002), platelet (PLT) (*ρ* = −0.327/*p* < 0.001), and lymphocyte (LYMP) levels (*ρ* = −0.216/*p* = 0.004). Total Ig levels positively correlated with ALT (*ρ* 0.288/*p* < 0.001), LDH (*ρ* = 0.207/*p* = 0.005), ferritin (*ρ* = 0.199/*p* = 0.008), WBC, neutrophils (NEU), PLT, and LYMP levels (*ρ* = 0.257/*p* = 0.001, *ρ* = 0.259/*p* < 0.001, *r* = 0.262/*p* < 0.001, and *r* = 0.161/*p* = 0.031), respectively.

Subsequent pooled analyses of the data by disease severity showed that, in the group of patients with mild-moderate disease, the SARS-CoV-2 Ag was inversely correlated with total Ig levels (*ρ* = −0.442/*p* = 0.001), PLT (*ρ* = −0.439/*p* = 0.002), ferritin (*ρ* = −0.386/0.006), and ALT (*ρ* = −0.386/*p* = 0.006). Total Ig levels positively correlated with WBC (*ρ* = 0.329/*p* = 0.021), LYMP (*ρ* = 0.430/*p* = 0.002), ferritin (*ρ* = 0.402/*p* = 0.004), and ALT (*ρ* = 0.338/*p* = 0.017). A significant negative correlation was observed between the total Ig levels and D-D (*ρ* = −0.411/*p* = 0.003), as well as lactic acid levels (*ρ* = −0.288/*p* = 0.045).

In the group of severe-critical cases, the SARS-CoV-2 Ag was inversely correlated with the levels of total Ig (*ρ* = −0.440/*p* < 0.001), WBC (*ρ* = −0.177/*p* = 0.044), LYMP (*ρ* = −0.252/*p* = 0.004), PLT (*ρ* = −0.283/*p* = 0.001), and ALT (*ρ* = −0.346/*p* < 0.001). The SARS-CoV-2 Ag positively correlated with CRP (*ρ* = 0.201/*p* = 0.022), lactic acid (*ρ* = 0.271/*p* = 0.002), and urea levels (*ρ* = 0.240/*p* = 0.006). Total Ig levels positively correlated with WBC (*ρ* = 0.191/*p* = 0.029), NEU (*ρ* = 0.253/*p* = 0.004), PLT (*ρ* = 0.331/*p* < 0.001), and D-D (*ρ* = 0.176/*p* = 0.044). A significant negative correlation was observed between the total Ig and creatinine levels (*ρ* = −0.298/*p* = 0.001).

**Figure 2:**
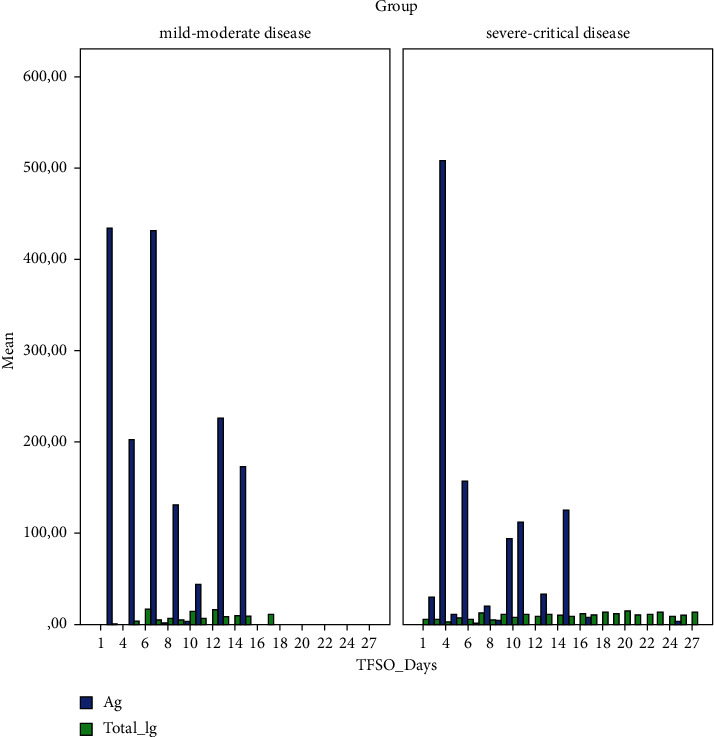
Mean of NP Ag and anti-S antibody titres over time in mild-moderate and severe-critical cases. TFSO : time from symptom onset (days).

**Figure 3:**
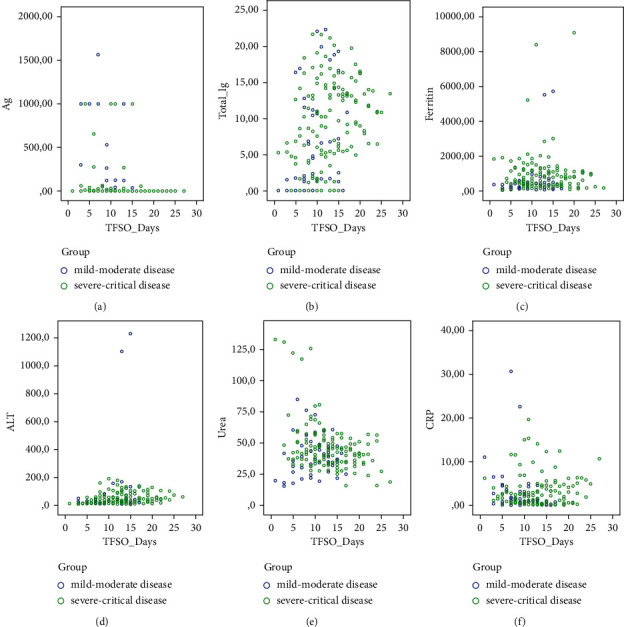
Values of the SARS-CoV-2 Ag (a), total Ig (b), ferritin (c), ALT (d), urea (e), and CRP (f) variables over time, in mild-moderate and severe-critical cases. TFSO : time from symptom onset (days).

The means of the NP Ag and anti-S total Ig titres over time by group are presented in Figure 2. The values of the SARS-CoV-2 Ag, anti-S total Ig, ferritin, ALT, urea, and CRP over time in mild-moderate and severe-critical cases are demonstrated in Figure 3.

### 3.3. Comparisons of Demographic and Clinical Characteristics between Mild-Moderate Cases and Severe-Critical Cases

Age and duration of symptoms from onset until admission did not differ significantly between the two groups. The frequency of hypertension was significantly lower in patients with mild-moderate disease as compared to the severe-critical cases (*p* = 0.044). The presence of nausea at admission was more common in mild-moderate cases compared with the severe-critical cases (*p* = 0.038).

### 3.4. Comparisons of the SARS-CoV-2 NP Ag, Anti-S Total Ig Levels, and Laboratory Values between Mild-Moderate and Severe-Critical Cases

Compared with mild-moderate cases, severe-critical cases had lower SARS-CoV-2 NP Ag levels (*p* = 0.002) and increased ferritin (*p* < 0.001), CRP (*p* = 0.040), urea (*p* = 0.005), and ALT (*p* = 0.004). Patients with severe-critical disease were also observed to have higher anti-S total Ig levels (*p* = 0.011) (mean rank comparisons, Table 2).

## 4. Discussion

In this study, we investigated the temporal patterns of the SARS-CoV-2 NP Ag and anti-S total Ig levels in hospitalized COVID-19 patients. Our results indicate a significant negative correlation between the SARS-CoV-2 NP Ag and anti-S total Ig levels regardless of the severity of COVID-19. It has been evidenced that IgG and IgM detection probabilities increase from roughly 10% at symptom onset to 98–100% by day 22 while RNA detection probability decreases from roughly 90% to zero by day 30 [11]. Our findings support the abovementioned report.

We also observed a strong dependence of specific laboratory markers with the SARS-CoV-2 NP Ag and anti-S total Ig levels. Several studies have commented on the hematological changes, especially lymphopenia and thrombocytopenia, that occur during the course of SARS-CoV-2 infection [12]. In our cohort, low WBC, lymphocyte, and PLT counts were associated with increased SARS-CoV-2 Ag levels. Previous research on SARS-CoV has suggested that the lung damage in SARS patients may induce thrombocytopenia either by increasing the consumption of megakaryocytes-platelets or by reducing the production of PLTs in the lungs [13].

Although no association was found between the SARS-CoV-2 NP Ag and neutrophil levels, rises in neutrophils positively correlated with anti-S total Ig levels. SARS-CoV-2-related neutrophilia has been described as an indicator of severe respiratory symptoms and poor prognosis [14, 15]. It has been shown that, in COVID-19 patients, circulating and lung-infiltrating neutrophils release higher levels of neutrophil extracellular traps (NETs) which induce the apoptosis of lung epithelial cells, thus contributing to the pathophysiology of severe COVID-19 [16]. Furthermore, it has been suggested that genetic factors affecting NETs may be involved in the disparity of COVID-19 severity [17].

Of note, when we analyzed our data by group, we observed that the anti-S total Ig levels positively correlated with PLT and neutrophil counts only in patients with severe-critical disease and not in mild-moderate cases. Moreover, in the latter group of patients, the anti-S total Ig levels inversely correlated with D-dimers while a positive correlation was observed between the two variables in severe-critical cases. These results are suggestive of distinct temporal associations between the anti-S total Ig levels with the values of the aforementioned hematological parameters in severe and critically ill patients. Similar conclusions, for the same group of patients, emerged for the SARS-CoV-2 NP Ag temporal profile in relation to the WBC and lymphocyte levels.

With respect to the biochemical parameters, we have previously reported the inverse correlation of ALT serum levels with the SARS-CoV-2 NP Ag [10], a finding which is reinforced in this study irrespective of patient categorization. The anti-S total Ig levels positively correlated with ALT, LDH, and ferritin in the initial pooled analysis, yet different results were obtained by the group analysis which also showed differences between the two groups in the temporal associations of the SARS-CoV-2 Ag and total Ig levels with creatinine, CRP, lactic acid, and urea. As in the case of hematological parameters, the temporal correlation patterns of biochemical markers with the SARS-CoV-2 NP Ag and anti-S total Ig levels varied depending on the severity of COVID-19.

Subsequently, we observed an excess of hypertension in severe and critical cases. It has been reported that underlying comorbidity, such as myocardial injury and cardiovascular disease, may relate to the mechanism by which hypertension leads to increased risk from COVID-19 [18]. Older age, uncontrolled blood pressure, and cardiovascular disease are thought as the three interconnected variables which could explain the observed associations between age, hypertension, and COVID-19 severity [18].

In our cohort, nausea at admission was most commonly reported by patients with mild-moderate disease. Recently, a study provided evidence that COVID-19 severity is significantly reduced in patients presenting with gastrointestinal symptoms when compared to those without gastrointestinal symptoms and that each separate symptom (nausea, vomiting, and diarrhea) is associated with less severe disease and lower mortality [19].

We also observed that, in comparison to mild-moderate cases, severe-critical cases had lower NP Ag and higher anti-S total Ig levels. In the literature, there is a substantial controversy regarding the prognostic importance of the SARS-CoV-2 viral load [4, 5, 20–24]. Since SARS-CoV-2 Ag quantification is indicative of the viral load, our findings support the notion that subjects with severe and critical disease have lower viral loads compared to those with mild and moderate disease. It would be of great interest to assess the correlation of the NP Ag with viral load in this study; however, data for viral load were not available for all time points. Our results with respect to the anti-S total Ig levels are in line with the inference of an excessive anti-SARS-CoV-2 antibody production which dampens protective immune responses in patients with severe COVID-19 [5].

Furthermore, our findings suggest that specific laboratory markers ferritin, ALT, urea, and CRP may be indicative of COVID-19 severity although large prospective studies are needed for identifying these variables as predictor factors. Several research groups have reported significant higher levels of ferritin in severe COVID-19 patients [25, 26]. Intestinal infection with SARS-CoV-2 has been associated with mild inflammatory response possibly due to the induction of potent neutralizing IgA antibodies which are mainly produced in the intestines [19]; thus, the underlying pathophysiological mechanisms of COVID-19-associated liver dysfunction should further be studied in the context of hepatocyte immune function [10].

It has been reported that, by using the machine learning method, research centers could establish accurate prediction models of outcome of SARS-CoV-2 pneumonia based on their laboratory findings [27]. Such a model recently identified CRP, LDH, and urea as significant prognostic factors for severe COVID-19 illness [28]. CRP, LDH, and D-dimer have also been associated with increased mortality risk in patients with comorbidities [29], while D-dimer and urea, in combination with troponin, have been identified as predictors of admission to the intensive care unit [30].

In accordance with our results, severe cases were found to have higher levels of antibody response and larger numbers of inflammatory cells and CRP levels in a study assessing the longitudinal dynamic changes in antibody responses against SARS-CoV-2 in 84 COVID-19 hospitalized patients [31].

Our study has some limitations. Αlthough NP is considered an ideal target for antigen-based detection [32], further research for the clinical validation of the lateral flow assay that we used is required. In addition, our cohort size was small and time from symptom onset to hospitalization varied greatly between patients. Larger datasets are needed so as to evaluate with greater confidence the kinetics of the SARS-CoV-2 Ag and total Ig responses during the clinical course of COVID-19. Despite these constraints, in this study, we performed repeated measurements of SARS-CoV-2 and host-related variables, thus providing insight into viral/host temporal dynamics in relation to the severity of COVID-19. Comprehensive information on virus and host kinetics during hospitalization and knowledge of SARS-CoV-2 seroconversion patterns [33] are important for the evaluation of therapy effectiveness and for clinical outcome improvement.

## 5. Conclusions

Distinct temporal profiles of the SARS-CoV-2 NP Ag and anti-S total Ig levels may distinguish different groups of COVID-19 severity.

## Figures and Tables

**Table 1:** Baseline characteristics of the study population.

	All patients (*N* = 40)	Mild-moderate cases (*N* = 12)	Severe-critical cases (*N* = 28)	*p* values
Age, years, median (range)	59.5 (30–90)	56.5 (30–87)	59.5 (34–90)	ns
Time from symptom onset to hospitalization, days, median (range)	8 (1–18)	6.5 (1–15)	8.5 (1–18)	ns
Male sex, *n* (%)	31 (77.5%)	8 (66.7%)	23 (82.1%)	ns
Positive smoking history, *n* (%)	16 (40%)	5 (41.7%)	11 (39.3%)	ns

*Comorbidities*				
Hypertension, *n* (%)	24 (60%)	4 (33.3%)	20 (71.4%)	0.024
Dyslipidemia, *n* (%)	12 (30%)	2 (16.7%)	10 (35.7%)	ns
Diabetes, *n* (%)	11 (27.5%)	1 (8.3%)	10 (35.7%)	ns
Coronary disease, *n* (%)	9 (22.5%)	1 (8.3%)	8 (28.6%)	ns
Obesity, *n* (%)	8 (20%)	1 (8.3%)	7 (25%)	ns
Immunosuppression, *n* (%)	4 (10%)	1 (8.3%)	3 (10.7%)	ns
Chronic obstructive pulmonary disease, *n* (%)	3 (7.5%)	1 (8.3%)	2 (7.1%)	ns
Any malignancy, *n* (%)	2 (5%)	1 (8.3%)	1 (3.6%)	ns
Chronic renal disease, *n* (%)	1 (2.5%)	0 (0%)	1 (3.6%)	ns
Other comorbidities, *n* (%)	27 (67.5%)	8 (66.7%)	19 (67.9%)	ns

*Symptoms at admission*				
Fever, *n* (%)	39 (97.5%)	12 (100%)	27 (96.4%)	ns
Shortness of breath, *n* (%)	22 (55%)	5 (41.7%)	17 (60.7%)	ns
Cough, *n* (%)	21 (52.5%)	6 (50%)	15 (53.6%)	ns
Weakness, *n* (%)	14 (35%)	5 (41.7%)	9 (32.1%)	ns
Arthralgia, *n* (%)	8 (20%)	4 (33.3%)	4 (14.3%)	ns
Headache, *n* (%)	5 (12.5%)	2 (16.7%)	3 (10.7%)	ns
Nausea, *n* (%)	4 (10%)	3 (25%)	1 (3.6%)	0.038
Chest pain, *n* (%)	3 (7.5%)	1 (8.3%)	2 (7.1%)	ns
Diarrhea, *n* (%)	3 (7.5%)	0 (0%)	3 (10.7%)	ns
Confusion, *n* (%)	1 (2.5%)	0 (0%)	1 (3.6%)	ns

*Complications at admission*				
Pneumonia, *n* (%)	34 (85%)	9 (75%)	25 (89.3%)	ns
Acute kidney damage, *n* (%)	7 (17.5%)	1 (8.3%)	6 (21.4%)	ns
Acute respiratory distress syndrome, *n* (%)	3 (7.5%)	0 (0%)	3 (10.7%)	ns
Cardiovascular damage, *n* (%)	2 (5%)	0 (0%)	2 (7.1%)	ns
Multiple organ failure, *n* (%)	2 (5%)	0 (0%)	2 (7.1%)	ns

**Table 2:** Descriptive statistics of the SARS-CoV-2 and host-associated variables that were serially measured in this study.

	Mean ± std. error of mean		*p* values^*∗*^V^*∗∗*^
	Mild-moderate cases	Severe-critical cases	
Ag (A.U.)	166.63 ± 52.85	43.51 ± 15.98	0.002
Total Ig (A.U.)	6.98 ± 1.02	9.51 ± 0.51	0.011
White blood cells (number/mm^3^)	8263.27 ± 479.54	8592.37 ± 259.11	ns
Neutrophils (number/mm^3^)	6410.41 ± 423.51	6593.36 ± 251.32	ns
Lymphocytes (number/mm^3^)	1193.47 ± 88.14	1136.34 ± 54.61	ns
Platelets (number/mm^3^)	297265.31 ± 17308.34	295992.37 ± 10156.99	ns
Ferritin (ng/ml)	539.77 ± 153.76	948.17 ± 103.89	<0.001
C-reactive protein (mg/dl)	2.85 ± 0.78	3.18 ± 0.31	0.040
D-dimer (ng/ml)	392.06 ± 37.14	417.82 ± 23.78	ns
Lactate dehydrogenase (U/L)	249.06 ± 10.69	267.20 ± 8.26	ns
Lactic acid (mmol/L)	0.87 ± 0.04	0.92 ± 0.02	ns
Urea (mg/dl)	38.74 ± 2.23	46.48 ± 1.74	0.005
Creatinine (mg/dl)	0.78 ± 0.03	0.89 ± 0.03	ns
Alanine transaminase^*∗∗*^ (U/L)	80.78 ± 32.76	51.28 ± 3.43	0.004

^*∗*^Mann–Whitney *U* test; ^*∗∗*^the mean ranks for Ag, total Ig, ferritin, C-reactive protein, urea, and ALT in mild-moderate cases were 105.43; 74.42; 57.50; 77.43; 72.84; and 72.29 versus 84.92; 96.52; 102.84; 95.39; 97.11; and 97.31 in severe-critical cases, respectively.

## Data Availability

The data that support the findings of this study are available upon request from the corresponding author.

## References

[1] George P. M., Barratt S. L., Condliffe R. (2020). Respiratory follow-up of patients with COVID-19 pneumonia. *Thorax*.

[2] Hasan S. S., Capstick T., Ahmed R. (2020). Mortality in COVID-19 patients with acute respiratory distress syndrome and corticosteroids use: a systematic review and meta-analysis. *Expert Review of Respiratory Medicine*.

[3] Iba T., Connors J. M., Levy J. H. (2020). The coagulopathy, endotheliopathy, and vasculitis of COVID-19. *Inflammation Research*.

[4] Fajnzylber J., Regan J., Coxen K. (2020). SARS-CoV-2 viral load is associated with increased disease severity and mortality. *Nature Communications*.

[5] Combes A. J., Courau T., Kuhn N. F. (2021). Global absence and targeting of protective immune states in severe COVID-19. *Nature*.

[6] Post N., Eddy D., Huntley C. (2020). Antibody response to SARS-CoV-2 infection in humans: a systematic review. *PloS One*.

[7] Giannoglou D., Meimeti E., Provatopoulou X. (2020). Predictors of mortality in hospitalized COVID-19 patients in Athens, Greece. *medRxiv*.

[8] Maltezou H. C., Raftopoulos V., Vorou R. (2021). Association between upper respiratory tract viral load, comorbidities, disease severity, and outcome of patients with SARS-CoV-2 infection. *The Journal of Infectious Diseases*.

[9] Wei P. F. (2020). Diagnosis and treatment protocol for novel coronavirus pneumonia (trial version 7). *Chinese Medical Journal*.

[10] Rouka E., Kotsiou O. S., Perlepe G., Pagonis A., Pantazopoulos I., Gourgoulianis K. I. (2021). Alanine aminotransferase serum levels in COVID-19 patients inversely correlate with SARS-CoV-2 antigen. *European Journal of Gastroenterology and Hepatology*.

[11] Borremans B., Gamble A., Prager K. C. (2020). Quantifying antibody kinetics and RNA detection during early-phase SARS-CoV-2 infection by time since symptom onset. *Elife*.

[12] Letícia de Oliveira Toledo S., Sousa Nogueira L., das Graças Carvalho M., Romana Alves Rios D., de Barros Pinheiro M. (2020). COVID-19: review and hematologic impact. *Clinica Chimica Acta*.

[13] Yang M., Li C., Li K. (2004). Hematological findings in SARS patients and possible mechanisms (Review). *International Journal of Molecular Medicine*.

[14] Wang J., Jiang M., Chen X., Montaner L. J. (2020). Cytokine storm and leukocyte changes in mild versus severe SARS‐CoV‐2 infection: review of 3939 COVID‐19 patients in China and emerging pathogenesis and therapy concepts. *Journal of Leukocyte Biology*.

[15] Cavalcante-Silva L. H. A., Carvalho D. C. M., Lima É. d. A. (2021). Neutrophils and COVID-19: the road so far. *International Immunopharmacology*.

[16] Veras F. P., Pontelli M. C., Silva C. M. (2020). SARS-CoV-2-triggered neutrophil extracellular traps mediate COVID-19 pathology. *Journal of Experimental Medicine*.

[17] Thierry A. R. (2020). Host/genetic factors associated with COVID-19 call for precision medicine. *Precis Clin Med*.

[18] Clark C. E., McDonagh S. T. J., McManus R. J., Martin U. (2021). COVID-19 and hypertension: risks and management. A scientific statement on behalf of the British and Irish Hypertension Society. *Journal of Human Hypertension*.

[19] Livanos A. E., Jha D., Cossarini F. (2021). Intestinal host response to SARS-CoV-2 infection and COVID-19 outcomes in patients with gastrointestinal symptoms. *Gastroenterology*.

[20] Carrasquer A., Peiró ÓM., Sanchez-Gimenez R. (2020). Lack of association of initial viral load in SARS-CoV-2 patients with in-hospital mortality. *The American Journal of Tropical Medicine and Hygiene*.

[21] Hasanoglu I., Korukluoglu G., Asilturk D. (2021). Higher viral loads in asymptomatic COVID-19 patients might be the invisible part of the iceberg. *Infection*.

[22] Néant N., Lingas G., Le Hingrat Q. (2021). Modeling SARS-CoV-2 viral kinetics and association with mortality in hospitalized patients from the French COVID cohort. *Proceedings of the National Academy of Sciences*.

[23] Kelleni M. T. (2021). SARS CoV-2 viral load might not be the right predictor of COVID-19 mortality. *Journal of Infection*.

[24] Le Borgne P., Solis M., Severac F. (2021). SARS‐CoV‐2 viral load in nasopharyngeal swabs in the emergency department does not predict COVID‐19 severity and mortality. *Academic Emergency Medicine*.

[25] Lin Z., Long F., Yang Y., Chen X., Xu L., Yang M. (2020). Serum ferritin as an independent risk factor for severity in COVID-19 patients. *Journal of Infection*.

[26] Gandini O., Criniti A., Ballesio L. (2020). Serum ferritin is an independent risk factor for acute respiratory distress syndrome in COVID-19. *Journal of Infection*.

[27] Wu G., Zhou S., Wang Y. (2020). A prediction model of outcome of SARS-CoV-2 pneumonia based on laboratory findings. *Scientific Reports*.

[28] Wu G., Yang P., Xie Y. (2020). Development of a clinical decision support system for severity risk prediction and triage of COVID-19 patients at hospital admission: an international multicenter study. *European Respiratory Journal*.

[29] Chen Z., Zhang F., Hu W. (2021). Laboratory markers associated with COVID-19 progression in patients with or without comorbidity: a retrospective study. *Journal of Clinical Laboratory Analysis*.

[30] Hachim M. Y., Hachim I. Y., Naeem K. B., Hannawi H., Salmi I. A., Hannawi S. (2020). D-Dimer, troponin, and urea level at presentation with COVID-19 can predict ICU admission: a single centered study. *Frontiers of Medicine*.

[31] Feng X., Yin J., Zhang J. (2021). Longitudinal profiling of antibody response in patients with COVID-19 in a tertiary care hospital in beijing, China. *Frontiers in Immunology*.

[32] Diao B., Wen K., Zhang J. (2021). Accuracy of a nucleocapsid protein antigen rapid test in the diagnosis of SARS-CoV-2 infection. *Clinical microbiology and infection : The Official Publication of the European Society of Clinical Microbiology and Infectious Diseases*.

[33] Murrell I., Forde D., Zelek W. (2021). Temporal development and neutralising potential of antibodies against SARS-CoV-2 in hospitalised COVID-19 patients: an observational cohort study. *PloS One*.

